# Comparing Risk Factors for Primary Multidrug-Resistant Tuberculosis and Primary Drug-Susceptible Tuberculosis in Jiangsu Province, China: A Matched-Pairs Case-Control Study

**DOI:** 10.4269/ajtmh.13-0717

**Published:** 2015-02-04

**Authors:** Xin-Xu Li, Wei Lu, Rong-Qiang Zu, Li-Mei Zhu, Hai-Tao Yang, Cheng Chen, Tao Shen, Guang Zeng, Shi-Wen Jiang, Hui Zhang, Li-Xia Wang

**Affiliations:** National Center for Tuberculosis Control and Prevention, Chinese Center for Disease Control and Prevention, Beijing, People's Republic of China; Center for Disease Control and Prevention of Jiangsu Province, Nanjing, People's Republic of China; Chinese Field Epidemiology Training Program, Chinese Center for Disease Control and Prevention, Beijing, People's Republic of China

## Abstract

To find out the reason why some people get infected directly with multidrug-resistant tuberculosis (MDR-TB), whereas some get infected with drug-susceptible tuberculosis (DS-TB), a 1:1:1 matched-pairs case-control study was conducted to identify predictors associated with primary MDR-TB and primary DS-TB against the control in Jiangsu Province, China. All three groups were geographically matched (by neighborhood) and matched on sex and age (±5 years). In total, 110 participants were enrolled in each of three matched groups. Conditional logistic regression analysis showed that predictors independently associated with primary MDR-TB were illiteracy or primary school education, annual per capita income ≤ US$2,000, per capita living space < 40 m^2^, and interval ≥ 7 days of eating fruits; predictors with primary DS-TB were body mass index ≤ 20 and feeling higher life pressure. This indicates that there are different predictors impacting the transmission range of primary MDR-TB and primary DS-TB in the general population.

## Background

Multidrug-resistant tuberculosis (MDR-TB), which is defined as TB caused by strains of *Mycobacterium tuberculosis* (MTB) that are resistant to at least isoniazid (INH) and rifampicin (RFP), is a form of TB infection linked with high morbidity and mortality, and it is of great importance to the National TB Control Program (NTP). According to the World Health Organization (WHO) report, China accounted for about 22% of the estimated 390,000–510,000 cases of incident MDR-TB (primary and acquired) arising globally in 2008.^1^ MDR-TB results from primary infection with resistant bacteria (primary MDR-TB), or it may develop in the course of a patient's treatment (acquired MDR-TB).^1^ The nationwide anti-TB drug resistance (DR) survey in China revealed the proportions of MDR-TB to be 5.7% (95% confidence interval [95% CI] = 4.6–7.1) in primary TB cases and 25.6% (95% CI = 21.7–30.0) in previously treated TB cases in 2007, which were higher than global levels of 2.7% (95% CI = 2.4–3.8) and 18.5% (95% CI = 14.2–31.7), respectively.^1^ These data suggested that the proportion of primary MDR-TB among primary TB cases is far lower than that of acquired MDR-TB among previously treated TB cases. However, the primary MDR-TB should not be ignored. China notified 869,092 primary TB cases and 54,216 retreatment TB cases in 2010,^2^ which indicates that estimated primary MDR-TB cases from primary TB cases are more than three times as many as acquired MDR-TB cases from retreatment TB cases.

Many surveys around the world have been conducted to identify risk factors for MDR-TB. Previous treatments for TB as a risk factor associated with MDR-TB were found in some surveys conducted in Brazil, Burkina Faso, Georgia, Spain, and Turkey.^3^–^7^ Other risk factors were previous use of fluoroquinolone and an injectable agent (other than streptomycin) in India,^8^,^9^ an initial treatment regimen that did not follow national guidelines in India,^9^ and treatment with INH and RFP for more than 180 days in China.^10^ Several studies showed that MDR-TB was closely associated with TB treatment, although it may also be related to other risk factors, such as sex,^5^,^11^ age,^6^,^10^,^11^ and human immunodeficiency virus (HIV).^11^ A survey in China also showed that retreatment TB patients were 5.48 times more likely to have MDR-TB than newly diagnosed TB patients.^10^ However, it is still unknown, in the general population without history of TB treatment and HIV, why some people get infected directly with MDR-TB, whereas some people get infected with drug-susceptible TB (DS-TB).

As noted above, despite lower prevalence of primary MDR-TB from primary TB cases than that of acquired MDR-TB from retreatment TB cases, the absolute numbers of primary MDR-TB cases estimated are larger than those of acquired MDR-TB cases. We have to hypothesize that there exists some risk factors associated with primary MDR-TB that are different from risk factors associated with primary DS-TB, although a survey in Peru found a high rate of primary MDR-TB in a general population with no identifiable risk factors.^12^ Therefore, the first step in our study was to extensively explore risk factors from sociodemographic characteristics, living environment, dietary patterns, daily life, etc., associated with primary MDR-TB in the general population and then, compare these risk factors with risk factors associated with primary DS-TB in the general population to seek differences in the high-risk populations for primary MDR-TB and primary DS-TB.

## Materials and Methods

### Estimation of sample size.

By using the formula of sample size for a 1:1 matched-pairs case-control study,^13^ the required sample size was estimated with the following assumptions: (1) odds ratio (OR) of importance ≥ 2.0, (2) a two-sided 95% CI, and (3) 90% power. Under these assumptions, the number of participants required to satisfactorily address the hypothesis was at least 90 cases and 90 controls.

### Participants' recruitment.

**Figure 1.**
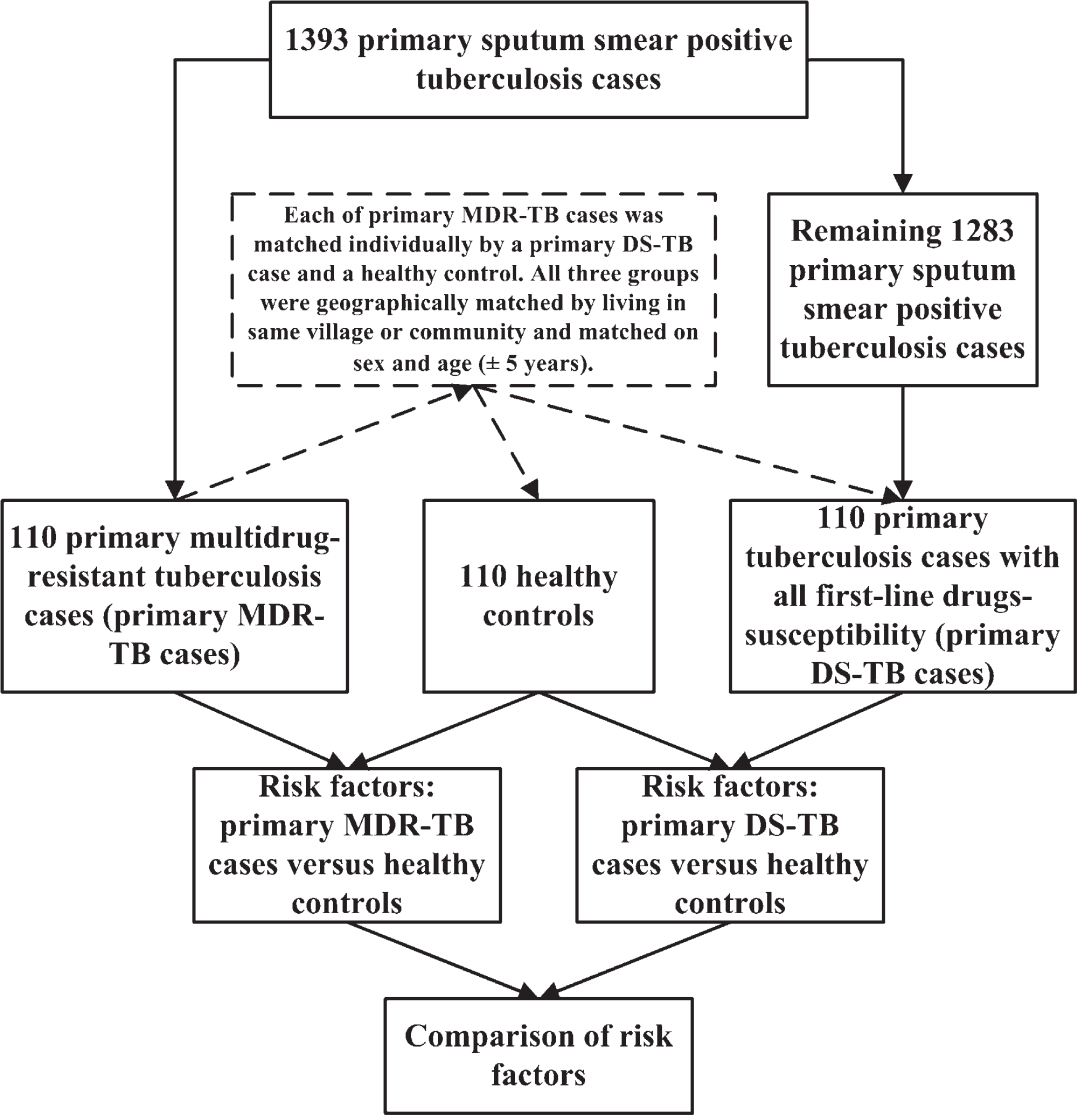
The study procedures.

From June to December of 2011, a 1:1:1 matched-pairs case-control study was conducted to identify risk factors associated with primary MDR-TB and primary DS-TB versus controls in Jiangsu Province, China. The laboratory procedures included two steps: first, suspected TB cases were diagnosed with TB mainly by sputum smear microscopy and chest imaging according to the guidelines for implementing the NTP in China,^14^ and second, MTB in bodies of TB cases was identified as MDR-TB or DS-TB using sputum culture and drug susceptibility testing (DST) with the proportion method for INH, RFP, ethambutol and streptomycin according to the guidelines for DR surveillance in TB (2nd edition) published by the WHO.^15^ Through the procedures above, in total, 110 primary MDR-TB cases were identified from 1,393 primary sputum smear-positive (SS+) TB cases by Disease Control and Prevention of Jiangsu Province (Jiangsu CDC). All 1,393 SS+ TB cases were distributed in 30 cities and counties of the whole province where the population density is 767 people/km^2^, one-half of whom came from rural settings. Each of the primary MDR-TB cases was matched individually with a primary DS-TB case and a control. The primary DS-TB case was specially defined as the primary SS+ case who was susceptible to all first-line drugs mentioned in this study. All three groups were geographically matched by living in the same village or community and matched on sex and age (± 5 years). The matched primary DS-TB cases were selected from the remaining 1,283 primary SS+ TB cases. The controls who were selected through the local population management system were not confirmed with TB or other pulmonary diseases according to diagnostic criteria of the NTP.^14^ The matched primary DS-TB cases and controls were included if they had the most similar matching conditions to primary MDR-TB cases. All participants were systematically tested for HIV. The study procedures can be seen in Figure 1.

### Participant's questionnaire.

To explore risk factors associated with primary MDR-TB and primary DS-TB, we used a structured questionnaire covering sociodemographic characteristics, living environment, dietary patterns, daily life, mental status, past medical history, and other potential risk factors in the year before they became MDR-TB or DS-TB cases, which were markers of socioeconomic status and general health status. All data were collected by self-reporting, except height and weight. The height and weight were collected from previous medical records or physical examination records of participants. The questionnaire was pre-tested before the study participants were interviewed. All of the interviews were conducted in participants' homes or rooms of local CDCs or TB dispensaries according to the participants' demands. The matched participants were interviewed on the same day by the same interviewer. Privacy was ensured while interviews were being conducted.

### Ethics statement.

This study was approved by the Ethics Review Committee (ERC) of Jiangsu Province CDC and the ERC of China CDC. Before the interviews, all participants provided their written informed consent. If participants were less than 18 years of age, their guardians provided the written informed consent.

### Statistical analysis.

For descriptive statistics, medians and interquartile ranges were calculated for continuous variables, and frequencies and percentages were calculated for discrete variables. The rank-sum test and the Pearson χ^2^ test were used for paired comparison of characteristics of three groups according to Bonferroni correction. Compared with control, risk factors with crude and adjusted matched ORs and their 95% CIs associated with primary MDR-TB and primary DS-TB were identified by univariate and multivariate conditional logistic regression models, respectively. A two-sided *P* value of 0.05 or less was regarded as the significance that was assessed by the Wald χ^2^ test. All data were analyzed using the SAS statistical package (version 9.2; SAS Institute, Inc., Cary, NC).

## Results

It is displayed in Table 1 that 110 primary MDR-TB cases, 110 matched primary DS-TB cases, and 110 controls were enrolled in the study. All the participants were HIV-negative. The median age of the three groups was all 48 years old, and 64.6% of each group was male. The body mass index (BMI) values in primary DS-TB cases were significantly different from those in controls. The proportions of those who were illiterate or had primary school education and those who felt higher life pressure in controls were significantly different from those in primary MDR-TB cases and primary DS-TB cases. Proportions of those who lived below the second floor in primary MDR-TB cases were significantly different from those in controls. Interval days of eating fruits in primary MDR-TB cases were significantly different from those in primary DS-TB cases and controls. Except those noted above, other characteristics were similar among the three groups.

According to Table 2, the univariate analysis showed that, compared with controls, risk factors significantly associated with primary MDR-TB were BMI ≤ 20 (OR = 4.33; 95% CI = 1.78–10.53), illiteracy or primary school education (OR = 3.75; 95% CI = 1.72–8.18), annual per capita income ≤ US$2,000 (OR = 5.33; 95% CI = 2.23–12.75), per capita living space < 40 m^2^ (OR = 2.86; 95% CI = 1.56–5.25), living below the second floor (OR = 3.80; 95% CI = 1.89–7.63), interval ≥ 7 days of eating fruits (OR = 3.75; 95% CI = 1.72–8.18), interval ≥ 2 days of physical exercise (OR = 4.00; 95% CI = 1.64–9.78), and feeling higher life pressure (OR = 3.60; 95% CI = 1.79–7.25). The multivariate analysis showed that risk factors independently associated with primary MDR-TB were illiteracy or primary school education (OR = 3.64; 95% CI = 1.37–9.70), annual per capita income ≤ US$2,000 (OR = 5.32; 95% CI = 1.22–23.22), per capita living space < 40 m^2^ (OR = 4.65; 95% CI = 1.06–20.38), and interval ≥ 7 days of eating fruits (OR = 6.10; 95% CI = 1.70–21.88).

As shown in Table 3, the univariate analysis showed that, compared with controls, risk factors significantly associated with primary DS-TB were BMI ≤ 20 (OR = 9.00; 95% CI = 3.20–25.28), illiteracy or primary school education (OR = 4.67; 95% CI = 1.93–11.27), per capita living space < 40 m^2^ (OR = 3.50; 95% CI = 1.60–7.68), interval ≥ 2 days of eating meats (OR = 5.00; 95% CI = 1.71–14.62), and feeling higher life pressure (OR = 6.50; 95% CI = 2.27–18.62). The multivariate analysis showed that risk factors independently associated with primary DS-TB were BMI ≤ 20 (OR = 11.10; 95% CI = 1.59–47.50) and feeling higher life pressure (OR = 6.31; 95% CI = 1.08–36.79).

## Discussion

Although MDR-TB is initially associated with irregular anti-TB treatment,^3^–^10^ the person-to-person transmission of MDR-TB gets more and more common. Many studies in the 1990s indicated that nosocomial transmission of MDR-TB happened, and health workers or HIV/acquired immune deficiency syndrome (AIDS) patients were the easily infected groups.^16^–^19^ Household contact, one of the MDR-TB transmission models, was documented in Brazil, Peru, India, and South Africa, and these studies showed that incidence rate of MDR-TB among household contacts was high; young children were also likely the victims.^20^–^24^ The transmission of MDR-TB during airplane flight was also observed.^25^ It is confirmed that these primary MDR-TB cases contacted the sources of infection; in other words, they are MDR-TB cases.

However, our results were contrary to the specific transmissions and groups noted above. We found in this study that primary MDR-TB cases, primary DS-TB cases, and controls had similar proportions of prior exposure to TB cases, which were similar with findings of a study in Peru and another study in China.^12^,^26^ This finding indicated that, among the general population with similar risks of exposure to TB (including DS-TB and MDR-TB), some people got infected with DS-TB, some people got infected directly with MDR-TB, and other people were not infected with any tubercle bacillus. Therefore, what factors caused the existence of the differences? What would the differences suggest for transmission range of MDR-TB? What measures would we have to take for prevention of primary MDR-TB?

This study attempted to extensively explore the possible clues to above-mentioned problems. There were only few studies of risk factors for primary MDR-TB, although many studies of risk factors for MDR-TB were found.^3^–^12^ In this study, we found that the independent risk factors for primary MDR-TB were different from primary DS-TB in the multivariate analysis, although some risk factors were similar in the univariate analysis. The independent risk factors associated with primary MDR-TB included poor education, low income, small living space, and less fruit consumption, and those associated with new DS-TB included low BMI and great psychological pressure.

Except less fruit consumption, other risk factors for primary MDR-TB in our study were involved in risk factors for MDR-TB in previous studies.^3^–^11^ It is easy to understand that those with poor education, low income, and small living space more likely developed primary MDR-TB cases, because infectious disease and poverty often go hand in hand. However, additional studies are required to find more information about less fruit consumption, such as the deficiencies of vitamins or trace elements in the body that are associated with primary MDR-TB; despite that, poor education, low income, and small living space more likely accompany unbalanced dietary patterns.^27^ Low BMI and great psychological pressure were not associated with primary MDR-TB in our study, whereas it was known that both of these risk factors had influence on the development of TB.^28^,^29^

Poor education, low income, and small living space as the external factors of society orient toward lower socioeconomic groups, whereas low BMI and great psychological pressure as the internal factors of an individual cannot specifically orient toward a social group. This maybe indicate that, compared with risk factors of primary DS-TB, primary MDR-TB more likely happens in the low social group. Previous studies displayed that the extensive transmission of MDR-TB had been indicated by molecular epidemiology and happened in urban and rural areas.^30^–^32^ However, our findings suggested that transmission range of MDR-TB is more limited than that of DS-TB and perhaps, still confined to the low social group.

The WHO advocated that rapid DST of both INH and RFP at the time of diagnosis is the most cost-effective strategy for any TB patient group or setting.^33^ However, because of plenty of suspected TB cases in China, surveillance for MDR-TB of all cases is impractical.^2^ Surveillance for MDR-TB among the suspected TB cases with high-risk factors associated with MDR-TB or those who live in MDR-TB transmission hotspots is a cost-effective technical method to control transmission of MDR-TB.^34^ Our findings can provide useful information for this in China. Additionally, our findings suggest that improving the social supports and living standards of the low social group may be the comprehensive strategy to control transmission of MDR-TB.

Several limitations of this study should be considered in the interpretation of the results. First, we obtained information about the year before participants became DS-TB or MDR-TB cases, and therefore, recall biases were unavoidable and potentially affected the results. Second, some information, such as annual per capita income, per capita living space, interval days of eating foods or physical exercise, and prior exposure to TB cases, might be inaccurate, because it was collected by self-reporting. Despite these limitations, the risk factors associated with primary MDR-TB may provide important information for additional studies in China.

## Conclusions

This matched-pairs case-control study identified risk factors associated with primary MDR-TB in China, which included poor education, low income, small living space, and less fruit consumption. They are different from risk factors associated with primary DS-TB, which are low BMI and great psychological pressure. It may be inferred from these findings that, compared with risk factors of DS-TB, the transmission range of MDR-TB is not very extensive and perhaps still confined to the low social group. Additionally, these findings can provide useful information for controlling transmission of MDR-TB.

## Figures and Tables

**Table 1:** Characteristics of primary MDR-TB cases, primary DS-TB cases, and controls in Jiangsu Province, China

Variables	Primary MDR-TB *n* (%)/median (IQR)	Primary DS-TB *n* (%)/median (IQR)	Control *n* (%)/median (IQR)	*P* value*
Total	110 (100.0)	110 (100.0)	110 (100.0)	
Sociodemographics				
Male	71 (64.6)	71 (64.6)	71 (64.6)	
Age (years)	48 (37, 59)	48 (30, 62)	48 (37, 58)	
BMI	21 (19, 25)	20 (19, 22)	23 (21, 24)	†
Illiteracy or primary school education	44 (40.0)	44 (40.0)	18 (16.4)	†‡
Single	37 (33.6)	37 (33.6)	21 (19.1)	
Staff/worker	34 (30.9)	39 (35.5)	41 (37.3)	
Migrant worker	32 (29.1)	21 (19.1)	32 (29.1)	
Farmer	23 (20.9)	28 (25.5)	25 (22.7)	
Annual per capita income (US$)	1,230 (564, 1,666)	993 (288, 2,230)	1,859 (800, 2,564)	
Living environments				
Per capita living space (m^2^)	26 (12, 54)	28 (19, 54)	40 (25, 60)	
Living below the second floor	78 (70.9)	64 (58.2)	46 (41.8)	‡
Dietary patterns				
Never eating three meals a day on time	9 (8.2)	14 (12.7)	3 (2.7)	
Interval days of eating meats	1 (1, 2)	1 (1, 4)	1 (1, 2)	
Interval days of eating coarse food grain	7 (1, 20)	2 (1, 18)	1 (1, 10)	
Interval days of eating fruits	3 (1, 25)	2 (1, 6)	1 (1, 3)	‡§
Daily behaviors				
Hours of sleep every day	8 (7, 9)	8 (7, 9)	8 (7, 9)	
Interval days of physical exercise	6 (3, 7)	6 (2, 7)	3 (1, 7)	
Feeling higher life pressure	44 (40.0)	39 (35.5)	14 (12.7)	†‡
Going to crowded fields at least one time per week	41 (37.3)	39 (35.5)	32 (29.1)	
Smoking	39 (35.5)	53 (48.2)	48 (43.6)	
Drinking alcohol	55 (50.0)	50 (45.5)	64 (58.2)	
Other potential factors				
Never vaccinating BCG vaccine	48 (43.6)	57 (51.8)	39 (35.5)	
Prior exposure to TB cases	48 (43.6)	60 (54.5)	46 (41.8)	

BCG = bacillus Calmette–Guérin; IQR = interquartile range.

* Bonferroni correction.

† *P* value < 0.0167 for primary DS-TB versus control.

‡ *P* value < 0.0167 for primary MDR-TB versus control.

§ *P* value < 0.0167 for primary MDR-TB versus primary DS-TB.

**Table 2:** Univariate and multivariate conditional logistic regression models of risk factors associated with primary MDR-TB

Variables	Univariate analysis			Multivariate analysis		
	Wald χ^2^ value	*P* value	Crude OR (95% CI)	Wald χ^2^ value	*P* value	Adjusted OR (95% CI)
Sociodemographics						
BMI ≤ 20	5.24	0.0221	4.33 (1.78–10.53)	0.31	0.5748	1.61 (0.30–8.60)
Illiteracy or primary school education	5.52	0.0188	3.75 (1.72–8.18)	6.70	0.0096	3.64 (1.37–9.70)
Single	3.70	0.0544	4.50 (0.97–13.30)	1.59	0.2076	2.90 (0.55–15.16)
Staff/worker vs. unemployed	1.69	0.1940	0.38 (0.09–1.64)	2.06	0.1513	0.44 (0.15–1.34)
Migrant worker vs. unemployed	0.98	0.3210	0.48 (0.11–2.04)	0.54	0.4644	0.64 (0.19–2.13)
Farmer vs. unemployed	1.32	0.2507	0.33 (0.05–2.20)	1.38	0.2402	0.42 (0.10–1.79)
Annual per capita income ≤ US$2,000	7.08	0.0078	5.33 (2.23–12.75)	4.93	0.0263	5.32 (1.22–23.22)
Living environments						
Per capita living space < 40 m^2^	5.71	0.0168	2.86 (1.56–5.25)	4.15	0.0417	4.65 (1.06–20.38)
Living below the second floor	7.05	0.0079	3.80 (1.89–7.63)	1.10	0.2953	2.15 (0.51–9.00)
Dietary patterns						
Never eating three meals a day on time	1.54	0.2150	4.00 (0.45–18.84)	0.41	0.5196	2.78 (0.12–32.67)
Interval ≥ 2 days of eating meats	0.04	0.8350	1.09 (0.48–2.47)	0.01	0.9435	0.98 (0.53–1.82)
Interval ≥ 3 days of eating coarse food grain	0.33	0.5655	1.40 (0.44–4.41)	0.16	0.6849	1.54 (0.19–12.42)
Interval ≥ 7 days of eating fruits	5.52	0.0188	3.75 (1.72–8.18)	7.71	0.0055	6.10 (1.70–21.88)
Daily behaviors						
≤ 7 hours of sleep every day	0.04	0.8350	1.09 (0.48–2.47)	1.30	0.2545	0.48 (0.13–1.71)
Interval ≥ 2 days of physical exercise	4.61	0.0317	4.00 (1.64–9.78)	0.03	0.8540	1.20 (0.18–8.06)
Feeling higher life pressure	6.42	0.0113	3.60 (1.79–7.25)	2.24	0.1340	1.97 (0.81–4.77)
Going to crowded fields at least one time per week	0.98	0.3226	1.67 (0.61–4.59)	0.74	0.3904	1.46 (0.62–3.48)
Smoking	0.79	0.3744	0.67 (0.27–1.63)	0.15	0.6999	1.32 (0.32–5.36)
Drinking alcohol	0.98	0.3226	0.60 (0.22–1.65)	0.20	0.6509	0.71 (0.16–3.18)
Other potential factors						
Never vaccinating BCG vaccine	0.87	0.3499	1.57 (0.61–4.05)	0.06	0.8051	1.20 (0.29–5.00)
Prior exposure to TB cases	0.04	0.8475	1.08 (0.51–2.29)	0.27	0.6054	0.72 (0.21–2.51)

BCG = bacillus Calmette–Guérin.

**Table 3:** Univariate and multivariate conditional logistic regression models of risk factors associated with primary DS-TB

Variables	Univariate analysis			Multivariate analysis		
	Wald χ^2^ value	*P* value	Crude OR (95% CI)	Wald χ^2^ value	*P* value	Adjusted OR (95% CI)
Sociodemographics						
BMI ≤ 20	8.69	0.0032	9.00 (3.20–25.28)	5.89	0.0152	11.10 (1.59–47.50)
Illiteracy or primary school education	5.86	0.0155	4.67 (1.93–11.27)	3.48	0.0623	7.84 (0.90–38.37)
Single	3.70	0.0544	4.50 (0.97–13.30)	0.70	0.4038	0.43 (0.06–3.10)
Staff/worker vs. unemployed	2.12	0.1450	0.29 (0.06–1.52)	0.44	0.5075	0.65 (0.18–2.31)
Migrant worker vs. unemployed	3.16	0.0752	0.20 (0.04–1.18)	2.41	0.1203	0.34 (0.09–1.33)
Farmer vs. unemployed	1.18	0.2781	0.36 (0.06–2.29)	0.86	0.3532	0.50 (0.11–2.18)
Annual per capita income ≤ US$2,000	2.77	0.0960	2.14 (0.87–5.25)	0.25	0.6154	1.44 (0.35–5.96)
Living environments						
Per capita living space < 40 m^2^	4.88	0.0271	3.50 (1.60–7.68)	3.24	0.0717	2.45 (0.92–6.51)
Living below the second floor	3.02	0.0825	2.33 (0.90–6.07)	0.75	0.3856	0.36 (0.04–3.56)
Dietary patterns						
Never eating three meals a day on time	2.75	0.0972	6.00 (0.72–26.79)	3.09	0.0786	6.92 (0.80–59.71)
Interval ≥ 2 days of eating meats	4.32	0.0377	5.00 (1.71–14.62)	3.28	0.0701	6.21 (0.57–37.41)
Interval ≥ 3 days of eating coarse food grain	0.05	0.8275	1.10 (0.47–2.59)	1.10	0.2934	0.36 (0.06–2.40)
Interval ≥ 7 days of eating fruits	0.59	0.4417	1.50 (0.53–4.21)	0.54	0.4604	1.70 (0.42–6.93)
Daily behaviors						
≤ 7 hours of sleep every day	1.17	0.2800	0.62 (0.26–1.48)	0.19	0.6617	0.75 (0.20–2.78)
Interval ≥ 2 days of physical exercise	1.11	0.2920	1.80 (0.60–5.37)	0.41	0.5199	1.64 (0.37–7.31)
Feeling higher life pressure	6.07	0.0137	6.50 (2.27–18.62)	4.19	0.0406	6.31 (1.08–36.79)
Going to crowded fields at least one time per week	0.36	0.5495	1.27 (0.58–2.80)	0.54	0.4614	1.78 (0.38–8.31)
Smoking	0.22	0.6380	1.25 (0.49–3.17)	0.28	0.5951	1.48 (0.35–6.34)
Drinking alcohol	1.92	0.1657	0.50 (0.19–1.33)	0.47	0.4909	0.58 (0.12–2.76)
Other potential factors						
Never vaccinating BCG vaccine	2.77	0.0960	2.14 (0.87–5.25)	0.52	0.4721	0.44 (0.05–4.11)
Prior exposure to TB cases	1.36	0.2436	1.60 (0.73–3.53)	3.50	0.0616	3.24 (0.94–11.11)

BCG = bacillus Calmette–Guérin.
